# Gender-related issues in a Taiwanese university medical science laboratory setting: a qualitative analysis

**DOI:** 10.3389/fpsyg.2023.1178921

**Published:** 2023-06-13

**Authors:** Chun-Yi Tseng, Shu-Ching Chang

**Affiliations:** ^1^College of Medicine, School of Medicine, Chang Gung University, Taoyuan City, Taiwan; ^2^Department of Medical Humanities and Social Sciences, College of Medicine, Chang Gung University, Taoyuan City, Taiwan; ^3^Department of Anatomic Pathology, Linkou Chang Gung Memorial Hospital, Taoyuan City, Taiwan

**Keywords:** higher education, gender equality, women researchers, scientific careers, health professional

## Abstract

**Introduction:**

This paper provides a glimpse into gender issues in a university-based medical science laboratory setting in northern Taiwan. In this study, gender issues with respect to perceptions regarding gender, the degree of gender neutrality in the work environment, and the influence of gender on researchers’ academic careers were analyzed.

**Methods:**

From July to August 2021, semistructured interviews to understand the perspectives of five faculty members at Chang Gung University School of Medicine regarding gender issues were conducted. The data were transcribed verbatim and analyzed thematically. Subsequently, coding was performed using ATLAS.ti Web (Version 4.0.10).

**Results and discussion:**

It was found that gender is not perceived to correlate with performance in the medical sciences. Although the medical science laboratories in the study institution are mostly gender-neutral, instances of discrimination might have been concealed elsewhere because of underreporting. Nevertheless, medical science research culture in Chang Gung University appears to promote respect and equality owing to increased general awareness regarding such issues as well as robust policies that protect women’s rights and promote gender equality. Marriage, motherhood, and family obligations remain key challenges to the academic career of female scientists in the institution. To achieve more equitable representation of male and female scientists as well as to prevent female scientists from leaving medical science laboratories in Taiwan, institutional and national policies that provide tailored support for female scientists intending to start families must continue to be implemented.

## 1. Introduction

There are 149 institutions of higher learning (universities, colleges, and institute of technology) in Taiwan as of 2021 (Higher Education Institution Numbers, 2023). Medical science is taught as a 4-year undergraduate course, with 8 private medical-based universities and 3 public comprehensive universities offering the most competitive programs. Although 47.59% of local medical science undergraduates choose to pursue master’s or doctorate programs after graduation (Taiwan Education Panel Survey and Beyond (TEPS-B) Newsletter, 2014), a disproportionately high percentage of women choose to quit the academic science workforce compared to men, with women accounting for 22.65% of researchers in Taiwan compared to 30.78% in Singapore and 38.60% in the United Kingdom (Department of Gender Quality, Executive Yuan, 2022). The “leaky pipeline” phenomenon proposed by writer and science journalist Natalie Angier in 1995 along with the “glass ceiling,” “sticky floor,” and “concrete walls” concepts described by sociologist Nancy Chodorow are indicators of immense resistance faced by female scientists climbing the academic ladder, particularly those aiming for senior faculty positions (Chodorow, 2012). The leaky pipeline phenomenon refers to the lower representation of women in academic positions, from the postdoctoral to associate and full professorship levels, despite the increasing number of doctorates being awarded to women (Montalvo et al., 2020).

**Figure 1:**
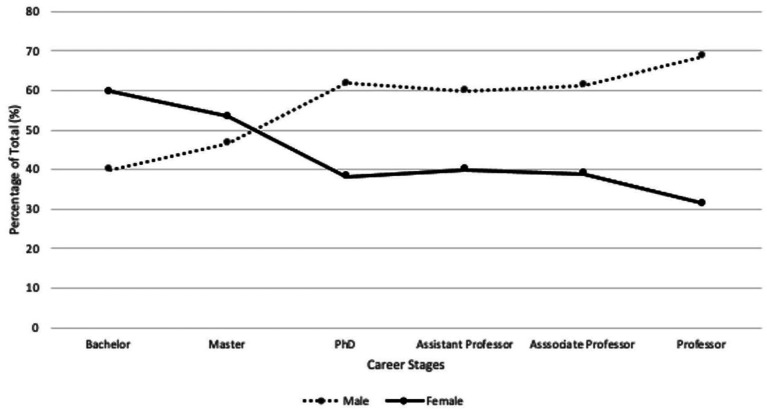
Career stage-based gender distribution of academics in Life Sciences in Taiwanese universities in 2019 (Database of gender makeup in Taiwan Higher Institutions, n.d.; Percentage of male and female students based on disciplines and academic level, n.d.). As defined by the Taiwan Ministry of Education, life sciences comprise biological science, ecology, biotechnology, microbiology, biochemistry, bioinformatics, and other sciences (genetics, bioengineering, neuroscience, fishery science, marine biology, food science, biological resources, and biophysics). Microbiology, and biochemistry are some examples of fields belonging to the medical sciences.

In a previous study, the career stage-based gender distribution of academics in the field of life sciences (including research fields under medical sciences) across higher education institutions in Taiwan was analyzed. The results revealed a scissor-shaped pattern of gender distribution (Figure 1) (Database of gender makeup in Taiwan Higher Institutions, n.d.; Percentage of male and female students based on disciplines and academic level, n.d.), similar to the trend observed in the education statistics of the 28 European Union (EU) member countries (EU-28) regarding the proportion of men and women in a typical academic career across all disciplines (European Commission, 2021). This evidence suggests that gender imbalance in science academia maybe widespread and underscores the significance of the issue. The “crossover point,” or the career stage in which the proportion of female academics in the medical sciences in Taiwan and the EU drops below that of male academics, occurs when an individual is considering to pursue a program at level 8 of the International Standard Classification of Education (i.e., at a doctoral or equivalent level).

Studies on gender disparities in science academia, especially focusing on the medical sciences, have indicated that gender bias can play a role in reducing the recruitment of women into laboratories staffed with elite male faculty members, thus depriving female scientists of key resources that facilitate professional development (Sheltzer and Smith, 2014; Montalvo et al., 2020). As a response to an increase in the incidents of gender inequity in educational campuses, the Taiwanese Government ratified the Gender Equity Education Act (GEEA) in 2004. The GEEA was established to promote “substantive gender equality, eliminate gender discrimination, uphold human dignity, and improve and establish education resources and environments for gender equality (Gender Equity Education Act, n.d.).” Gender equity training was mandated for all public and private school faculty and staff members across all education levels (Sinacore et al., 2019). For example, institutions are mandated by law to establish a Gender Equity Education Committee (GEEC), which is tasked to investigate reported incidents of bullying or gender discrimination as well as to design gender equity curriculum for students, staff, and faculty (Sinacore et al., 2019). However, a study conducted in 2022 reported that 56.97% of women in Taiwan still felt that gender discrimination is common in the workplace (Rich et al., 2022). Contemporary research conducted in higher education context in China has predominantly focused on exploring the factors that influence the intention to pursue higher education (Dai et al., 2021) or on analyzing the status of gendered education (Wang and Stocker, 2010; Si, 2022). A previous study identified gender norms as a contributor to the geographical immobility of Chinese women academics (Bao, 2022) and another study analyzed factors that prevent girls and women from pursuing a scientific career in Taiwan (Cheng, 2010). However, only a few studies from Taiwan have employed a qualitative approach, in which academic scientists in medical sciences were encouraged to share their experiences of working in laboratory settings.

Up to date knowledge is critical to gain a better understanding of contemporary gender climate in university-based research settings in Taiwan. Elucidating the research climate through a qualitative approach can help inform, update, and accelerate policies to increase female participation in senior academic positions. In this study, gender issues regarding perceptions of gender within the medical sciences work environment, and the effects of gender on researchers’ academic careers were analyzed. This study was performed with a university-based laboratory setting as the primary focus to understand research culture because it is the location in which most science academics spend a substantial amount of time conducting studies. For this study, faculty members were selected as they have the highest level of teaching experience and influence on the daily activities of a laboratory. Daily activities that underpin the research process, including those that contribute to the recruitment of students into a laboratory, can provide valuable insights into gender issues in the medical sciences.

## 2. Methods

A thematic analysis was performed by conducting semistructured interviews among faculty members at Chang Gung University (CGU) College of Medicine (COM) from July to August 2021.

CGU is a private university that is widely recognized in the field of clinical and medical sciences in Taiwan. CGU COM comprises 18 departments including medicine, nursing, physical therapy, and medical sciences. Faculty members across the departments work in areas such as clinical science, molecular medicine, and medical biotechnology. Medical sciences cover research fields pertaining to molecular biology, biochemistry, microbiology and immunology, parasitology, physiology, pathology, pharmacology, and anatomical sciences. Senior faculty members are principal investigators for their research teams. Laboratory was defined as a physical space that may house one or more research teams working in different fields. The gender distribution data of the faculty members in the CGU COM departments are presented in Table 1.

**Table 1:** Gender data of departments in CGU COM (June 2022).

Departments in CGU COM classified as basic medical science (*n* = number of faculty members with academic rank of assistant professor and above)	Number of faculty members with the academic rank of assistant professor and above (%)	
	Male participants	Female participants
Biochemistry and molecular biology (*n* = 11)	7 (63.6%)	4 (36.4%)
Anatomy (*n* = 7)	3 (42.9%)	4 (57.1%)
Microbiology and immunology (*n* = 9)	3 (33.3%)	6 (66.7%)
Physiology and pharmacology (*n* = 11)	5 (45.5%)	6 (54.5%)
Parasitology (*n* = 3)	2 (66.7%)	1 (33.3%)

### 2.1. Qualitative method and design

After considering the average research team size in CGU, faculty members who taught at COM with teams of 5 to 20 members, including bachelor, masters, and PhD students and research assistants, were recruited. In addition to conducting primary research, these faculty members teach courses at the undergraduate to graduate levels, supervise students, and are involved in administrative tasks and curriculum design. It was considered that laboratories with exceptionally large or small groups of members may not be representative of laboratory culture. The participants were selected through purposive sampling and invited to participated in the interview *via* email. The study protocol was approved by the Institutional Review Board of Chang Gung Medical Foundation. Prior to study commencement, written informed consent was obtained from all participants.

Each participant was assigned an identification code comprising a letter and a number (Table 2). To ensure participants’ anonymity, they are herein referred to by their identification codes. The letters “A” and “B” denote female and male participants, respectively. The numerical part of the code denotes the gender makeup of the faculty member’s research team. Specifically, “1″ indicates that the research team comprised more women than men, “2″ indicates that the ratio of women and men was equal, and “3″ indicates that the team had more men than women.

**Table 2:** Participant characteristics.

Identification code*	Academic rank	PhD (country)	Research field
A1	Associate professor	PhD (Taiwan)	Molecular biology
A2	Professor	PhD (United States)	Microbiology
A3	Professor	PhD (United States)	Immunology
B1	Professor	PhD (Taiwan)	Biochemistry
B3	Assistant professor	PhD (Taiwan)	Virology

* The letters “A” and “B” denote female and male participants, respectively. The numerical part of the code denotes the gender makeup of the faculty member’s research team; “1” indicates that the research team comprised more women than men, “2” indicates equal ratio of women and men, and “3” indicates that the team had more men than women.

Owing to coronavirus disease 2019 (COVID-19) restrictions, in-depth interviews were performed through Google Meet. Each interview lasted approximately 20–30 (mean: 28) min and was conducted in Chinese by the first author. The interviews were semistructured and consisted of predetermined, open-ended questions (Supplementary material) that had been sent to the participants beforehand. Each session was audiotaped and transcribed verbatim for coding and subsequent referencing. To ensure that thematic saturation was reached and that no critical information had been overlooked or omitted, the transcripts were sent to the participants for proofreading and content confirmation. Thereafter, the transcripts were read by the authors and open coding was performed. Common themes among the interviews were generated from the open codes. Subsequently, qualitative analysis, which involved generating codes and developing recurrent themes through the following six steps (Braun and Clarke, 2012), was performed: familiarization with the data, generation of preliminary codes, identification of themes, review of potential themes, designation of themes, and production of the final report. The data were independently read by the authors to familiarize with the content and context. Both descriptive and interpretive initial codes were generated. Coding was performed using ATLAS.ti Web (Version 4.0.10), a qualitative data analysis software. Thereafter, themes were developed by identifying similarities and areas of overlap in the codes. To ensure relevance, only themes that occurred consistently in all interviews were selected and subsequently analyzed. This process involved rigorous, extensive discussions to strengthen the credibility and reliability of the generated themes and to enable data triangulation. Before drafting the manuscript, it was ensured that each theme was assigned an appropriate label.

## 3. Results

### 3.1. Qualitative data and analysis

Of the five participants, 3 and 2 (60 and 40%, respectively) were women and men, respectively. The participants held academic ranks either equal to or above assistant professor. Their characteristics are presented in Table 2. According to the codes generated from the interviews, four themes were generated.

### 3.2. Gender is not perceived to correlate with performance in basic medical science

The participants believed that gender does not correlate with an individual’s competency or interest in basic medical science. When asked what qualities an exceptional researcher possesses, none mentioned gender as a crucial factor. However, some participants asserted that female students tend to have more patience and that male students tend to be more logical. Female faculty members interviewed indicated that personality, teamwork skills, and organizational skills are critical contributors to an individual’s performance. In addition to the above qualities, male faculty members emphasized on skills such as critical and logical thinking. All participants attributed factors other than gender and gender norms to explain female students’ preference for life sciences as opposed to other science, technology, engineering, and mathematics (STEM) fields, such as engineering, computer science, and mathematics (which are traditionally viewed as masculine disciplines).

Some boys might be stronger in their analytical skills; therefore, they might excel in bioinformatics. Girls can be equally skilled in this discipline. [Similarly,] success in medical science, which requires a student to be highly motivated, have grit, and think logically, is much dependent on personality traits and other individual characteristics. It has no relationship with one’s gender. (A2)

Unlike that in mathematics and physics, which requires strong logical thinking skills […], success in medical science requires critical thinking abilities and imagination owing to] the multidisciplinary nature of this field. (B3)

### 3.3. Gender issues in the laboratory might be concealed

The participants were highly aware of the need to uphold and promote gender equity and to promote a respectful work environment in the laboratory. They emphasized that physical stamina, a characteristic typically associated with masculinity, was not an essential quality that they looked for when selecting students. The nature of experiments in the biomedical sciences requires researchers to have a greater level of patience, a characteristic typically associated with femininity; for example, when a researcher is working with model organisms or waiting for microbiological culture results. However, the participants indicated that applicants are not preferentially selected according to their gender. Instead, the admission process involves a holistic assessment of the candidate through interviews and examination of their academic profile. Matters related to gender, such as inappropriate treatment of students by faculty staff or opposite genders and sexual harassment, are considered sensitive. The male faculty members who took part in this study indicated that they were especially cautious in this regard because any mismanagement of the aforementioned incidents can result in severe repercussions, tarnishing the reputation of a research team. This suggests that instances of unfair treatment might go undetected or undocumented should they occur. Moreover, the mechanisms underlying such incidents may operate insidiously.

For me, [a good] personality and the ability to work in a team matter the most. Spending a long time in the laboratory means that a student needs to have a sense of teamwork. Otherwise, friction and other problems will occur within the laboratory. (B1)

Recent incidents of sexual harassment have garnered public attention and caused widespread commotion. Thus, when approaching a topic, I tend to remind myself to tread carefully, including how I manage professional relationships between myself and my students as well as the relationships among my research students. (B3)

### 3.4. Medical science research environment in CGU seems to promote respect and equality

The female faculty members interviewed agreed that the research culture in the medical sciences was favorable. In the basic medical sciences, direct gender inequality is likely not among the factors responsible for the “leaky pipeline” and the scissor-shaped pattern of the distribution of academic researchers. In other words, workplace discrimination is unlikely to be the primary reason for the lower proportion of female senior faculty members in biomedical science-related fields despite women constituting a larger proportion of biomedical undergraduates. Factors such as maternity leave and family commitments were the most frequently noted reasons for female scientists’ departure from academia.

I felt that I was discriminated because of my lack of research experience in the United States. After returning to Taiwan, I never felt disrespected or discriminated because of my gender. (A2)

There is something called the old boys’ club in the United States […]. You might expect the research environment there to be more open than that in Taiwan, but this is not necessarily true. There might be insidious discrimination toward women and racial minorities. [Although] it is not apparent, you can feel it. (A3)

### 3.5. Marriage, motherhood, and family obligations probably remain key challenges for female scientists in Taiwan

All female faculty members interviewed in this study expressed that marriage, motherhood, and family obligations had the strongest influence on their academic roles. Motherhood typically requires a period of leave from the laboratory, potentially delaying research progress. Balancing career and family commitments, such as caring for young children, can result in unpredictability and logistical challenges. A successful career and a fulfilling family life are not necessarily mutually exclusive. Nonetheless, when their children are in their formative years, female scientists might be compelled to choose between professional and family-related responsibilities. Generally, marriage, motherhood, and family obligations are linked to the societal expectations for women in Sinitic cultures. The interviewed male faculty members acknowledged that family commitments might be a factor that affects a female scientist’s career.

Having a child and needing to invest time in raising a family made me feel like I was no longer in control of my life […], which was really challenging and tough. [However,] I believe that the majority of female academics are able to overcome this […]. It just requires systematic planning and making the most of the limited time you have. (A1)

For female scientists, the struggle has always been between family and career. There is a societal expectation that women need to take on the role of childrearing. It is inevitable that if you dedicate more time to your family, sacrifices will have to be made in terms of professional development and career advancement. (A3)

## 4. Discussion

Majority of gender studies have focused on gender gaps in STEM and several theories regarding the relatively low participation of women in have been proposed. One theory is that science is inherently masculine in its structure, epistemology, and methodology (Blickenstaff, 2005). Potential contributors to the gender imbalance in STEM research roles include a lack of role models, incongruency with gender roles and gender identity, and an unfriendly classroom environment (Jones et al., 2000). Proponents of biological determinism assert that female students tend to show greater interest in the biological sciences than in the physical sciences (e.g., physics and chemistry) (Sax et al., 2016; Kang et al., 2019).

In this milieu, physics, computer science, and engineering are typically perceived to be masculine fields, whereas the biological sciences (medical science and health), languages, and humanities are considered more feminine fields (Jones et al., 2000; van der Vleuten et al., 2016). Several studies have revealed sex differences in cognitive aptitude. For example, male students have been reported to outperform female students in spatial abilities, mechanical reasoning, and systemizing. Moreover, female students generally score higher in empathy, which might explain prevailing gender stereotypes regarding their supposed suitability for careers in biology and health-related subjects (Forgasz et al., 2014; van der Vleuten et al., 2016; Stewart-Williams and Halsey, 2021). Gender stereotypes reinforce gender-based differences in performance, which may have effectuated the prevailing notion that male and female students are more suited to the physical and biological sciences, respectively (Forgasz et al., 2014).

The participants of this study believed that biological sex is not perceived to be correlated with competence and performance in biomedical science and that competence and performance are, instead, considerably influenced by diligence, personality, and other qualities such as interpersonal and organizational skills. The higher proportion of women in undergraduate and master’s courses in medical science might not be the result of an innate predisposition. Instead, it may be an unintended consequence of social expectations and of medical science being represented as involving less mathematics, which may lead female students to view the field more positively (Weinburgh and Englehard, 1994). In another study, female scientists in traditionally male-dominated fields reported feeling obliged to engage in gender performance, by “acting like one of the boys” (Powell et al., 2009). Specifically, they reported of feeling a need to establish a favorable academic reputation for themselves by conforming to societal models of masculinity (Powell et al., 2009). However, despite the biological sciences being considered more feminine fields according to gender stereotypes, female researchers in the medical sciences continue to quit their workplaces. This phenomenon can be attributed to the weight of societal expectations of women as default caregivers and homemakers, and this is omnipresent for female scientists. Although striking a balance between work and family commitments is attainable, female scientists are consistently compelled to choose between the two. This does not indicate that having a successful career and a fulfilling family life are mutually exclusive; rather, it highlights the fact that female scientists’ decisions are constrained by social pressures and expectations.

From the responses collected in this study, awareness of gender equality in the study institution has benefited through the enactment of laws such as the GEEA. Other contributors to gender equality awareness throughout higher education institutions in Taiwan were accomplishments of women’s rights movements, such as the establishment of the Awakening Foundation, a nonprofit organization championing for gender equality in Taiwan, and the passing of the GEEA in 2000, which upholds the employment rights of married or pregnant women (Chiang and Liu, 2011). Other nonprofit organizations, such as the Society of Taiwan Women in Science and Technology, also helped to “promote women’s professional status in STEM fields (About TWiST [Internet], 2020).” As stipulated by the GEEA, instructors in educational institutions must attend one to two sessions of gender-related retraining courses each semester. Notably, as demonstrated by the male faculty members’ marked hesitation and careful deliberation when answering questions related to gender equality in the workplace, these sessions may have contributed to the heightened awareness of gender equality and the sensitivity to such challenges among academics.

The present study findings indicate that marriage, motherhood, and family obligations seem to continue to constitute key challenges and considerations for female scientists in the study institution. Female scientists surveyed in a study published in Scientific American noted that both motherhood and family roles such as childcare and household chores are substantial time commitments (Cole and Zuckerman, 1987). A study revealed that Sinitic higher education settings do not offer favorable conditions for women, who may be constrained by familial responsibilities and the social pressure of being a mom and wife (Sinacore et al., 2019). The Taiwanese Government launched policies in 2021 to support female researchers, particularly expectant mothers and mothers with children aged ≤3 years, with a grant from the Ministry of Science and Technology. Under these policies, female researchers were offered additional assistance in terms of assignment of research personnel and other staff members (Ministry of Science and Technology, 2021).

This paper provides a glimpse into the gender issues in a university-based medical science laboratory setting. The environments of these laboratories emphasize individual attributes such as personality, teamwork, and organizational skills while promoting respect. However, Taiwan’s unique cultural and sociopolitical history of women’s rights movements and policies enacted to protect women’s rights might have resulted in false reassurances and unfair treatment and discrimination being concealed. Policies addressing the challenges that marriage, motherhood, and family commitments potentially present might prevent female scientists from leaving medical science laboratories in Taiwan. Given the steady contributions of senior female scientists to COVID-19 pandemic control, the participation of women in medical science in Taiwan should continue to be promoted. Nevertheless, creating an environment that supports female scientists in Taiwan requires further effort, and more qualitative research at a larger scale is warranted to help understand how this population wishes to be supported in this context. This study might continue to draw attention to gender issues, especially in medical sciences higher education training, and facilitate conversation regarding the academic work environment in Taiwan.

This study had several limitations. First, this was a single-center study. The relatively small number of participants and focus on the basic medical sciences might affect the generalizability of the findings, including their applicability to other research institutions. Nevertheless, given that this study was conducted in a university well-known in the fields of general and clinical medicine, the findings contribute to the knowledge of current research climate in Taiwanese higher institutes of learning. Second, the study did not involve faculty members from any of Taiwan’s national universities or public research institutions. These faculty members generally receive substantially more funding than researchers in private universities. These differences might contribute to potential differences in the research climate. Third, only senior faculty members of CGU COM were interviewed and the perspectives of younger researchers or students were not consider. However, relative to their junior counterparts, senior faculty members may have more experience and insight regarding potential reasons for gender disparity in academia, including differences that emerge as researchers ascend the academic ladder. Senior faculty members are also more likely to have family commitments and to possess research experience in foreign institutions, which might have enriched the diversity and depth of the responses obtained regarding gender issues in laboratory settings.

## Data availability statement

The original contributions presented in the study are included in the article/Supplementary material, further inquiries can be directed to the corresponding author.

## Ethics statement

The studies involving human participants were reviewed and approved by Chang Gung Medical Foundation Institutional Review Board (IRB) at Chang Gung Memorial Hospital. The patients/participants provided their written informed consent to participate in this study.

## Author contributions

C-YT and S-CC contributed to the conception of the study, conducted the coding, critically analyzed the qualitative data, and revised the manuscript. C-YT conducted the interview and drafted the initial manuscript. All authors contributed to the preparation of the article and approved the submitted version.

## Funding

This work was supported by the Taiwan Ministry of Science and Technology (MOST 2813-C182-056-H and MOST 108-2511-H-182-013-MY3) and Linkou Chang Gung Memorial Hospital (BMRP961).

## Conflict of interest

The authors declare that the research was conducted in the absence of any commercial or financial relationships that could be construed as a potential conflict of interest.

## Publisher’s note

All claims expressed in this article are solely those of the authors and do not necessarily represent those of their affiliated organizations, or those of the publisher, the editors and the reviewers. Any product that may be evaluated in this article, or claim that may be made by its manufacturer, is not guaranteed or endorsed by the publisher.

## Abbreviations

CGU: Chang Gung University
COM: College of Medicine
EU: European Union
GEEA: Gender Equity Education Act
STEM: Science, technology, engineering, and mathematics
STEMM: Science, technology, engineering, mathematics, and medicine.
