# Supplementary material for: Gender-related issues in a Taiwanese university medical science laboratory setting: a qualitative analysis
Source: Front Psychol. 2023 Jun 13;14:1178921. doi: 10.3389/fpsyg.2023.1178921 (PMC10293633; doi:10.3389/fpsyg.2023.1178921)
Supplement: Supplementary file 1 [file Data_Sheet_1.docx]

Supplementary Material

Gender-Related Issues in a Taiwanese University Medical Science Laboratory Setting: A Qualitative Analysis

Chun-Yi Tseng, Shu-Ching Chang*

*** Correspondence:** Shu-Ching Chang: [d868404@mail.cgu.edu.tw](mailto:d868404@mail.cgu.edu.tw)

# Supplementary Data （Interview Questions）

1. Please introduce yourself and provide a brief overview of your academic qualifications, experience in research, primary research field, and area of interest.
2. Please describe the gender makeup of your laboratory. What do you think caused this gender difference in your laboratory and in the basic medical sciences in general?
3. What are some salient features that an ideal medical science researcher should possess?
4. When considering applications to join the laboratory (i.e., applications from undergraduate students seeking to complete a research project module or from postgraduate students completing a masters or PhD program), what are the attributes that matter most to you (e.g., experience or academic achievement)?
5. When guiding research students in the laboratory, do you guide male and female students differently?
6. Do male and female students exhibit any differences in their performances in the laboratory?
7. Other than the facilities and scope of the research, what do you think are the differences between medical science and STEM (Science, Technology, Engineering, and Mathematics) laboratories?
